# Relaxed Selection Drives a Noisy Noncoding Transcriptome in Members of the *Mycobacterium tuberculosis* Complex

**DOI:** 10.1128/mBio.01169-14

**Published:** 2014-08-05

**Authors:** Adam M. Dinan, Pin Tong, Amanda J. Lohan, Kevin M. Conlon, Aleksandra A. Miranda-CasoLuengo, Kerri M. Malone, Stephen V. Gordon, Brendan J. Loftus

**Affiliations:** ^a^ UCD Conway Institute, University College Dublin, Dublin, Ireland; ^b^ Wellcome Trust Cell Biology Centre, The University of Edinburgh, Edinburgh, United Kingdom; ^c^ UCD School of Veterinary Medicine, University College Dublin, Dublin, Ireland; ^d^ School of Medicine and Medical Science, UCD Conway Institute, University College Dublin, Dublin, Ireland

## Abstract

Related species are often used to understand the molecular underpinning of virulence through examination of a shared set of biological features attributable to a core genome of orthologous genes. An important but insufficiently studied issue, however, is the extent to which the regulatory architectures are similarly conserved. A small number of studies have compared the primary transcriptomes of different bacterial species, but few have compared closely related species with clearly divergent evolutionary histories. We addressed the impact of differing modes of evolution within the genus *Mycobacterium* through comparison of the primary transcriptome of *M. marinum* with that of a closely related lineage, *M. bovis*. Both are thought to have evolved from an ancestral generalist species, with *M. bovis* and other members of the *M. tuberculosis* complex having subsequently undergone downsizing of their genomes during the transition to obligate pathogenicity. *M. marinum*, in contrast, has retained a large genome, appropriate for an environmental organism, and is a broad-host-range pathogen. We also examined changes over a shorter evolutionary time period through comparison of the primary transcriptome of *M. bovis* with that of another member of the *M. tuberculosis* complex (*M. tuberculosis*) which possesses an almost identical genome but maintains a distinct host preference.

## IMPORTANCE

Our comparison of the transcriptional start site (TSS) maps of *M. marinum* and *M. bovis* uncovers a pillar of conserved promoters, noncoding RNA (NCRNA), and a genome-wide signal in the −35 promoter regions of both species. We identify evolutionarily conserved transcriptional attenuation and highlight its potential contribution to multidrug resistance mediated through the transcriptional regulator *whiB7*. We show that a species population history is reflected in its transcriptome and posit relaxed selection as the main driver of an abundance of canonical −10 promoter sites in *M. bovis* relative to *M. marinum*. It appears that transcriptome composition in mycobacteria is driven primarily by the availability of such sites and that their frequencies diverge significantly across the mycobacterial clade. Finally, through comparison of *M. bovis* and *M. tuberculosis*, we illustrate that single nucleotide polymorphism (SNP)-driven promoter differences likely underpin many of the transcriptional differences between *M. tuberculosis* complex lineages.

## INTRODUCTION

The genus *Mycobacterium* enumerates more than 100 species, among which are pathogens of global importance to both humans and livestock (1), including the causative agent of tuberculosis, *M. tuberculosis*. The genome of *M. tuberculosis* has been sequenced for over a decade, and yet many important issues relating to the biology of mycobacteria remain poorly understood. Despite the application of comparative genomics, there is a dearth of studies illustrating how evolutionary forces shape transcriptomes within this genus. *M. bovis*, the causative agent of bovine tuberculosis, contains 3,952 predicted protein-coding genes and shares >99.95% sequence identity with *M. tuberculosis* (2). *M. marinum* is a pathogen of amphibians and one of the species most closely related to the *M. tuberculosis* complex, sharing almost 3,000 orthologs with *M. bovis* and *M. tuberculosis* (ca. 75% of their total coding regions) (3). This core set of orthologs, with average amino acid identity of 85%, has underpinned the use of *M. marinum* as a model organism to study mycobacterial virulence and the evolution of the *M. tuberculosis* complex (3). The 6-Mb genome of *M. marinum* is appreciably larger than those of the members of the *M. tuberculosis* complex, which are thought to have undergone a process of reductive evolution as a consequence of niche adaptation to the mammalian host cell environment (4). Though their genomes lack the obvious signatures of genome degradation found in other obligate pathogens, members of the *M. tuberculosis* complex are characterized by low sequence diversity and clonal evolution (5, 6).

**TSS mapping.** Differential RNA sequencing (dRNA-seq) enriching for primary transcripts has emerged as a highly accurate means of mapping the primary transcriptomes of a number of species (7, 8). Transcriptional start site (TSS) mapping through delineation of the regions upstream of the translation start points (TSPs) facilitates the identification of promoters and 5′ untranslated region (5′ UTR)-associated regulatory elements across an entire genome. The discovery of copious numbers of TSSs not obviously associated with mRNA transcripts in a number of studies has led to an explosion in the numbers of predicted noncoding RNA (NCRNA) species. The very presence, however, of such large numbers of TSSs presents a significant challenge in identifying NCRNAs that are likely to act as regulatory elements, as only a small subset have been assigned functional roles. Conservation across conditions or between related species has been used as a surrogate for functionality in some studies (9) to account for the difficulty in interpreting the potentially confounding additive effects of evolutionary forces. However, when the primary evolutionary forces acting on species are expected to be similar, their effects on the transcriptome are difficult to clearly identify. Given that relaxed selection is thought to be a significant force in the *M. tuberculosis* complex lineage (10), we compared the primary transcriptome of *M. marinum* with that of a member of the *M. tuberculosis* complex (*M. bovis*). These organisms share a highly conserved core of orthologous genes that have experienced evolution in appreciably different ways, which should enable a comparison of the effects of reduced selection on transcriptional complexity.

## RESULTS

### Comparison of *M. marinum* and *M. bovis* reveals a core set of conserved promoters.

**FIG 1:**
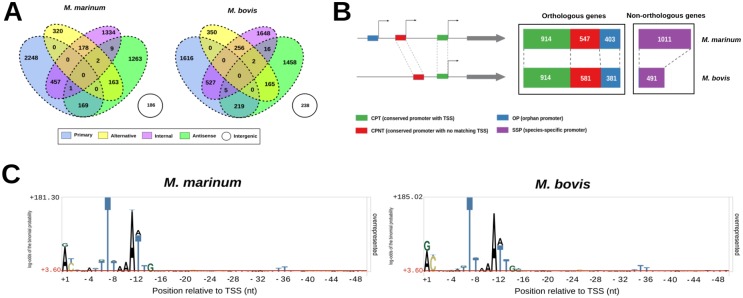
(A) Venn diagrams outlining the distribution of experimentally identified TSSs in *M. marinum* and *M. bovis* species. (B) Classification scheme used for TSSs associated with orthologous genes. Data represent the distribution of pTSSs associated with orthologous genes in each category for *M. marinum* and *M. bovis*. (C) pLogo depiction of base overrepresentation at nucleotide positions relative to the TSSs among conserved (CPT) primary promoters. The red horizontal lines indicate statistical significance, with Bonferroni correction. It can be seen that T residues at positions −35 and −36 are statistically enriched within these promoters relative to randomly selected genomic regions.

Whole-transcriptome sequencing was performed on two independent biological replicates of exponentially growing *M. bovis* AF2122/97 and *M. marinum* M. The pairwise correlation coefficients for these replicates demonstrated a high degree of reproducibility between runs (see Table S1 in the supplemental material). dRNA-seq libraries yielded ~12 to 13 million reads per replicate for both species, of which ~40% to 60% mapped to the reference genomes following removal of rRNA reads. Alignment of the terminator exonuclease (TEX)-positive (TEX^+^) and TEX^−^ dRNA-seq libraries to the genomes of *M. marinum* and *M. bovis* resulted in the identification of 6,331 and 6,500 individual TSSs, respectively (see Table S1). Genome-wide TSS maps were generated by categorizing TSSs depending on their position relative to annotated genes, in a manner similar to that described by Sharma et al. (7) (see Fig. S1 in the supplemental material). Specifically, all TSSs were assigned to at least one of five categories: primary TSS (pTSS), alternative TSS (altTSS), internal TSS (iTSS), antisense TSS (asTSS), and intergenic TSS (igTSS) (Fig. 1A). A full breakdown of the motifs found within the promoter regions of both species is given in Fig. S2.

A total set of 2,939 orthologous genes shared between *M. marinum* and *M. bovis* was identified for comparative analyses (see Table S1 in the supplemental material). A total of 1,730 of 2,875 (60%) pTSSs detected in *M. marinum* and 1,876 of 2,367 (79%) detected in *M. bovis* were assigned to genes classified as orthologous. This indicates that our analysis of regulatory regions is relevant for the majority of the shared transcriptomes under the conditions tested. To detect the presence of shared regulatory features, we analyzed the upstream regions of orthologous genes in both species, employing a strategy derived from a recent study comparing the transcriptomes of *Escherichia coli* and *Klebsiella pneumoniae* (11) (Fig. 1B).

Specifically, having defined a promoter as the 50-nucleotide (nt) region upstream of a TSS, we categorized all TSSs into one of four groups based on conservation of the promoter sequence and the presence or absence of a TSS in the other species. Category one (conserved promoter with TSS [CPT]) consisted of a conserved promoter region with a TSS called in both species. Category two (conserved promoter with no matching TSS [CPNT]) consisted of a conserved promoter with a TSS present in only one of the species. Category three, consisting of the orphan promoter (OP), showed no promoter conservation and a TSS in only one species, while category four promoters, the species-specific promoters (SSP), were promoters restricted to species-specific genes.

A total of 914 primary TSSs were classified as CPT (see Table S2 in the supplemental material) sharing conserved promoter regions, thus allowing a direct comparison of their associated regulatory regions. The 5′ UTR lengths of these genes, as defined by their primary TSS, were positively correlated (Pearson *r* = 0.55, *P* <2.2e-16), with 577 (63%) having lengths within 10 nt of each other (see Table S3). Utilizing data from a recent study by DeJesus et al. (12) that reassigned TSPs in *M. tuberculosis*, we found that the 5′ UTR length proved an accurate predictor of the direction and scale of corresponding reannotations for 66 of 80 (83%) of *M. bovis* orthologs within the CPT data set (see Table S3).

### MIRUs.

Where length differences were observed between the 5′ UTRs of orthologous genes, a subset could be attributed to the presence of mycobacterial interspersed repetitive units (MIRUs) in *M. bovis* 5′ UTRs (13). MIRU-based variable-number tandem-repeat (VNTR) molecular typing is a widely used tool for genotyping of clinical isolates of *M. tuberculosis*, and MIRU elements are hypothesized to be transcribed and to contribute to antigenic variability (14, 15). Additionally, studies have shown that VNTR copy number variants are capable of altering promoter activity in neighboring genes (15, 16). The extent of MIRU influence on transcriptional control has not been extensively studied; however, of the 24 MIRU loci used for VNTR typing, 17 overlap or are contained within *M. bovis* 5′ UTRs (see Table S3 in the supplemental material). For example, gene MMAR_1344, encoding the chromosome segregation protein ParA in *M. marinum*, was found to be transcribed in a leaderless fashion, with exactly overlapping TSS and TSP positions. However, the orthologous gene in *M. bovis* (*M. bovis* Mb3239c) harbors a 77-nt 5′ UTR consisting entirely of a class I MIRU. Notably, several regulatory genes carry MIRUs within their 5′ UTRs (see Table S3), suggesting that MIRUs may exert a wider transcriptional influence than has previously been appreciated.

### Identification of a conserved signal at the −35 region of mycobacterial promoters.

The consensus −10 hexamer motif (TANNNT), which is recognized by the sigma A (SigA) principle σ factor in mycobacteria, was the dominant transcriptional regulatory motif identified in both species (Fig. 1C), being found upstream of 50.3% of TSSs detected in *M. marinum* and 62.8% of TSSs detected in *M. bovis*. The extended −10 class of promoter (TGNTANNNT) was identified upstream of 2.7% of TSSs in *M. marinum* and 4.4% of TSSs in *M. bovis*. Similarly to a recently published data set from *M. tuberculosis* (17), promoters containing extended −10 motifs were found to be associated with significantly larger TSS peak heights in both species (*P* for both, <2.2e-16, Kruskal-Wallis test) (see Fig. S3 in the supplemental material). Among promoters lacking a −10 box, two alternative motifs were detected (see Fig. S2), and both were associated with lower TSS peak heights than the consensus −10 box (*P* <2.2e-16 in both species, Kruskal-Wallis test). The more prominent of the two alternative motifs harbors a consensus G residue at position −14 relative to the TSS [(−14)GNNANNNT(−7)]. Studies in *E. coli* suggest it is likely that a G residue at this position can compensate for the lack of a T residue at its canonical −12 position (18).

No single −35 hexamer has been identified across mycobacteria, and a recent global study of *M. tuberculosis* promoters was unable to identify a consensus motif at the site (17). However, the DNA sequence at this region can influence the activity of mycobacterial promoters by modulating recognition by RNA polymerase (RNAP), and mutation or inversion of the −35 area can have an impact on transcription. In order to search for a conserved −35 signal, we examined the promoters of the shared (CPT) primary TSSs from both species using the motif visualization package pLogo (19), which allows an assessment of the statistical significance of sequence features compared to a background data set (see Materials and Methods). pLogo identified the existence of thymine (T) residues occurring above the level of statistical significance (*P* < 0.05, with Bonferroni correction) at positions −35 and −36 in both species (Fig. 1C). Within the 914 CPT TSSs, 162 (18%) were found to share a conserved TT motif within the −35 region in both species (see Table S3 in the supplemental material). A number of the genes in this subset, including those encoding translation initiation factors (*infA* and *infC*), lipoproteins (*lpqD* and *lpqF*), and the WhiB family transcriptional regulators (*whiB1* and *whiB3*), are functionally related.

### Transcriptional attenuation within the 5′ UTRs of shared orthologs.

The 5′ ends of mRNA molecules are important *cis*-acting regulatory elements, which can mediate transcriptional attenuation in response to a variety of stimuli (20). These complex RNA structures sometimes harbor low-level primary-sequence conservation, and only a small proportion are likely to have been identified and annotated (20). Regulatory elements within 5′ UTRs may be revealed by the premature attenuation of transcription upstream of their associated genes, as indicated by high ratios of 5′ UTR-to-coding sequence (CDS) transcription, normalized to reads per kilobase per million mapped reads (RPKM) (21, 22).

We identified a total of 291 genes with long (≥30-nt) 5′ UTRs and high 5′ UTR-to-CDS RPKM ratios in *M. marinum* and 208 such genes in *M. bovis*, 56 of which were shared by the two species (see Table S3 in the supplemental material). The 5′ UTRs of genes within this subset had significantly higher sequence identities (median identity = 53%) than those of other orthologous genes (median identity = 41%) (*P* < 0.005, Wilcoxon Mann-Whitney test). Small regulatory RNAs (sRNAs) have been predicted in 7 of these 5′ UTRs in *M. tuberculosis* (23), while an additional 6 are predicted to contain structured RNAs with homologs in the Rfam database of RNA families. The evolutionary conservation of high 5′ UTR-to-CDS RPKM ratios provides support for the idea of the participation of 5′ UTRs in transcriptional attenuation (20).

For example, the gene *whiB7*, which is upregulated in response to a variety of antibiotics and regulates the expression of a number of proteins involved in intrinsic antibiotic resistance (24), was found to have prominent 5′ UTR expression relative to that of its CDS in both species (Fig. 4). Within the 5′ UTRs of *whiB7* in both species, we identified a small, unannotated upstream open reading frame (uORF) located ~10 nt downstream of a consensus Shine-Dalgarno (SD) motif. Transcription is seen to spike at the 5′ of the uORF and attenuates rapidly 3′ of the uORF, consistent with the presence of a conserved stem-loop structure with a T-rich tail, which may function as a rho-independent terminator (RIT) (Fig. 4). Previous studies have identified this ORF upstream of *whiB7* in *M. tuberculosis* and have shown that *whiB7* is transcriptionally coupled to it (25). Our data showing that the uORF and *whiB7* have been cotranscribed across evolutionary time provide a strong case for its involvement in the regulation of *whiB7* expression.

The expression levels of many genes are controlled by uORFs, which can respond to physiological signals (26). In the case of the *erm* family of macrolide resistance genes, antibiotic-promoted ribosome stalling and frameshifting in the uORF can independently activate antibiotic resistance regulons (27–29). A notable exception to this is the *erm*(37) of *M. tuberculosis*, which is part of the *whiB7* regulon (24), and macrolide resistance in mycobacteria results from the induction of *whiB7* (30). An intriguing possibility raised by our analysis is that antibiotic resistance in mycobacteria occurs via uORF-mediated transcriptional attenuation. Mutations in the 5′ UTR of *whiB7*, which can mediate antibiotic resistance in *M. tuberculosis* (31), may therefore promote translational readthrough of the uORF and result in activation of the *whiB7* regulon.

Other genes found to have high 5′ UTR-to-CDS RPKM ratios in both species included the *M. marinum* (MMAR_0811) and *M. bovis* (Mb0495) orthologs of the *M. tuberculosis* transcriptional regulator Rv0485, which regulates the *PE13* and *PPE18* gene pair and is essential for virulence (32). Similarly, the gene encoding the pyruvate dehydrogenase E1 subunit A (*pdhA*) component contains a highly expressed 5′ UTR relative to the CDS in both species, in agreement with a previous report in *Bacillus subtilis* indicating that the leader region may participate in regulation (33).

### Pervasive transcription initiation drives divergent distribution of TSSs between *M. marinum* and *M. bovis*.

**FIG 2:**
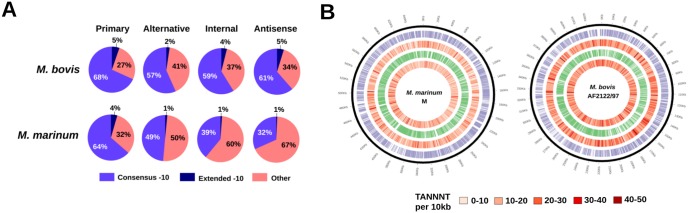
(A) Pie chart distribution of consensus −10 box motifs detected within the promoters across each TSS class between *M. marinum* and *M. bovis*. (B) Comparison of SigA consensus −10 box motif densities along the genomes of both species. Forward strand genes are indicated in purple, and reverse strand genes are in green.

A striking feature of the TSS maps of *M. marinum* and *M. bovis* is the divergence in TSS distribution, with *M. bovis* manifesting a much larger proportion of its assigned TSSs in all categories except the primary TSS (Fig. 1A). Additionally, in marked contrast to the relatively high conservation of pTSSs, other categories displayed an almost complete lack of conservation, with just 50 iTSSs and 49 asTSSs in total shared between the species, based on the best reciprocal alignment of the promoter sequences (see Table S2 in the supplemental material). A comparison of the contributing motifs across the different TSS classes reveals that the elevated distribution of nonprimary TSSs in *M. bovis* is driven by an expanded representation of −10 box consensus promoters in these classes relative to *M. marinum* (Fig. 2A).

We looked to determine whether this overrepresentation of −10 box promoters was driven by underlying differences in the sequence compositions of the genomes of the two species. Although the GC content of *M. marinum* (65.7%) is almost identical to that of *M. bovis* (65.6%), the latter has 15,569 consensus −10 box motifs (3,674 per Mb on average), a significantly higher density than that of *M. marinum*, with an average of 2,556 per Mb (*P* <2.2e-16, Pearson’s chi-square test) (Fig. 2B). The number of −10 box motifs places a theoretical upper limit on the level of SigA-driven transcription initiation and predicts that such initiation is theoretically possible at over 1,000 additional locations per Mb in *M. bovis* compared to *M. marinum*.

**FIG 3:**
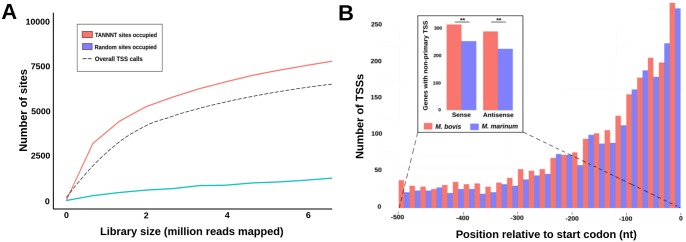
(A) Line plots show the number of −10 box motifs showing RNAP occupancy at the indicated levels of sequence depth in *M. bovis* compared with randomly selected genomic sites. The dashed line shows the total number of actual TSS calls made with the available sequence data at each depth. (B) Expansion of TSSs within the upstream regions of orthologous genes in *M. bovis*. A histogram shows the density of TSSs upstream of orthologs with conserved TSSs (CPTs) in both species. The inset shows the distribution of nonprimary TSSs located within 500 nt of the TSP in both species. **, *P* < 0.005, Pearson’s chi-squared test.

To explore the extent to which a genome’s density of −10 boxes correlates with promoter occupancy, we determined the number of consensus −10 sites in *M. bovis* being filled by at least 10 TEX^+^ reads, a number which is lower than the cutoff of 20 reads used for actual TSS calls, while maintaining a minimum TEX^+^/TEX^−^ ratio of at least 2:1. Applying this relaxed criterion, designed to mimic the effect of increased sequencing depth, resulted in 7,770 (50%) of the total 15,569 consensus −10 box sites being occupied at the fully mapped library size of ~6.6 million TEX^+^ reads (excluding rRNA). We also determined the degree to which −10 site occupancy by the accumulation of TEX^+^ reads contributes to TSS identification by mapping the relationship across different library sizes (Fig. 3A). The rate at which −10 boxes were found to become occupied mirrors almost exactly the call rate for TSSs, indicating that the identified TSSs and their distributions are largely functions of the presence of an appropriate initiation site combined with an appropriate sequencing depth. In contrast, equivalent numbers of randomly selected genomic positions show a significantly lower level of occupancy at each depth (*P* <2.2e-16, Pearson’s chi-square test).

**FIG 4:**
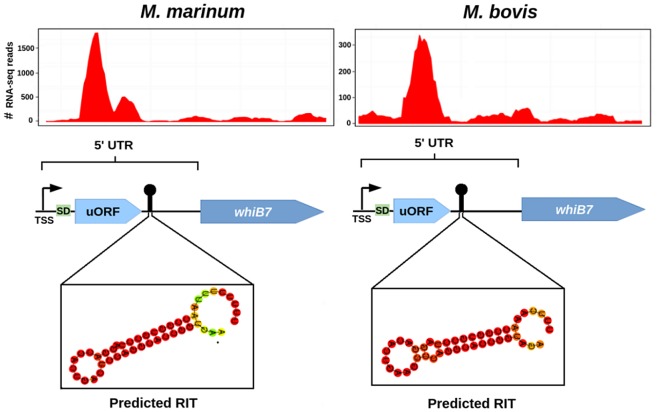
Conservation of attenuation patterns in the 5′ UTRs of *whiB7*. Transcription initiates at conserved primary TSSs located ~350 nt upstream of the *whiB7* start codons and peaks in both species within the indicated upstream ORFs (uORFs). Transcriptional attenuation then occurs at the 3′ ends of the uORFs, proximal to a conserved stem-loop structure followed by a U-rich stretch, predicted to function as a rho-independent terminator (RIT).

These results indicate that the various densities of TANNNT motifs can contribute to large-scale divergences in primary transcriptome composition, even between closely related mycobacterial species. As the mutational bias is toward AT in bacteria (34), a parsimonious interpretation of the enlarged noncoding transcriptome of *M. bovis* relative to *M. marinum* is that it is driven by an accumulation of A+T-enriching single nucleotide polymorphisms (SNPs) ineffectually purged by natural selection (35). As an extreme example, the genome of *M. leprae*, which has ~8% lower GC content than other mycobacteria and has experienced extensive genomic degradation (36), contains a very high density of consensus sites (12,728 per Mb, on average).

### Limited conservation of asTSSs between *M. marinum* and *M. bovis* belies a thematic conservation of antisense transcription.

Sequencing in *M. tuberculosis* has uncovered an abundance of asRNA, generally originating antisense to a coding region or from the 3′ UTR of a gene facing the opposite direction (21). The presence of pervasive intragenic transcription initiation in *M. bovis* relative to *M. marinum* is reflected in a very low level of conservation between the species, in line with data from other studies (9). However, in both species, the genes in the subset with high antisense-to-sense RPKM ratios (≥0.75) were enriched for insertion sequences and phages (*P* < 0.05, Pearson’s chi-square test), and this antisense transcription may serve to repress transposition. A role for asRNA in genome maintenance has been suggested in a previous study in the rapidly growing species *M. smegmatis* (37), indicating that this likely represents a conserved function of asRNA in mycobacteria. TSS-driven asRNA was identified opposite the Cas1 and Cas2 genes of the clustered regularly interspaced short palindromic repeat (CRISPR) locus in *M. bovis*, analogous to that reported for *M. tuberculosis* (21), suggesting another potential role for antisense transcription in genome defense.

### 5′ UTRs and conservation of alternative TSSs.

Our results indicate that each species has a degree of transcriptional “baggage” and predict that this phenomenon should affect all nonprimary TSS classes. pTSSs are assigned by peak height and proximity to a gene (≤500 nt), with altTSSs residing within the same region and having lower peak heights. To compare the TSS compositions of this region between the species, we analyzed the set of 914 orthologs with conserved pTSS (CPT). Overall, we found that *M. bovis* was more likely to have multiple TSSs upstream of these genes than *M. marinum* (*P* < 0.005, Pearson’s chi-square test) (Fig. 3B). Additionally, *M. bovis* was more likely to contain at least one nonprimary antisense TSS (i.e., an antisense TSS not associated with a divergently transcribed gene) within this region (*P* < 0.005, Pearson’s chi-square test) (Fig. 3B, bottom right). The increased transcriptional initiation occurring on both strands proximal to the pTSSs of CPT orthologs may reflect increased transcriptional complexity in *M. bovis* or, as seems more likely, may result from the influence of relaxed selection and genetic drift on the transcriptome of *M. bovis*.

### Intergenic sRNAs.

The genome-wide identification of TSSs has aided in the discovery and prediction of intergenic small regulatory RNAs (sRNAs), which can act in *trans* on their regulatory targets (38). These *trans*-encoded RNAs generally exist as short (~50-to-300-bp) transcripts capable of acting locally or globally and resulting in the formation of secondary structures that can affect mRNA stability or occlude ribosome binding (38). A number of studies in different mycobacteria have reported the identification of intergenic sRNAs (21, 23, 39–41). To determine the degree of overlap of our TSS maps, we assembled a list of 42 experimentally validated intergenic sRNAs from mycobacterial species (18 from *M. bovis* BCG [39, 41], 16 from *M. tuberculosis* [21, 23], 7 from *M. smegmatis* [37], and 1 from *M. avium* [40]) and mapped their locations by sequence homology to the genomes of both species (see Table S4 in the supplemental material).

In total, of the 42 sRNAs, 22 could be mapped unambiguously to locations within the *M. marinum* genome, while 41 could be mapped to *M. bovis*. Of these, 14 (64%) were assigned TSSs in the former in our study, while 21 (51%) were assigned TSSs in the latter. A total of 11 TSS-associated sRNAs were common to the two species, representing a core set shared between the lineages (see Table S4 in the supplemental material). Notably, 9 TSS-associated sRNAs (41%) in *M. marinum* and 12 (29%) in *M. bovis* were identified within regions annotated as 5′ UTRs, indicating the challenge of distinguishing 5′ regulatory elements from bona fide intergenic transcripts.

### Comparison of TSS maps identifies SNP-driven promoter differences between *M. bovis* and *M. tuberculosis*.

**FIG 5:**
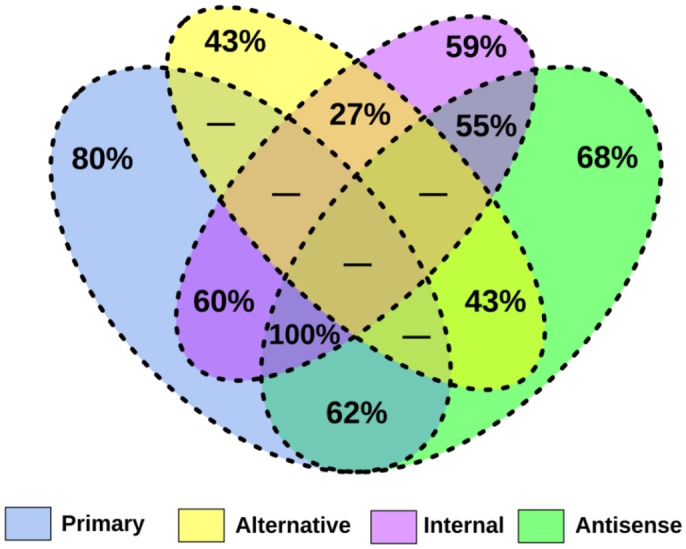
Overlap of the TSS maps of *M. bovis* and *M. tuberculosis*. For each TSS category, the percentage of potential overlap achieved is indicated. The overlap of pTSS is highest, with a majority of asTSSs and iTSSs also shared.

The transcriptional landscape of *M. tuberculosis* during exponential *in vitro* growth has recently been elucidated by Cortes et al. (17). *M. bovis* differs from *M. tuberculosis* in its host preference but possesses an almost identical genome and contains no unique genes. Given that differential gene expression may contribute to the molecular underpinnings of their differing phenotypes, we sought to determine the extent of overlap of the primary transcriptomes of *M. bovis* and *M. tuberculosis*. As the overall TSS numbers called for *M. bovis* were higher, we benchmarked the degree of conservation against TSSs identified in *M. tuberculosis* (Fig. 5). We found overall that a majority of the pTSSs, asTSSs, and iTSSs of the two data sets overlap (Fig. 5), while the level of overlap of altTSSs was lower.

It is likely that many of the TSSs detected only in *M. bovis* are also functional in *M. tuberculosis* but have not been annotated, perhaps due to the sequencing depth employed. Indeed, inspection of the TSS-seq data generated for *M. tuberculosis* (17) shows a significantly greater pileup of TEX^+^ reads at such positions than at randomly selected genomic sites (*P* <2.2e-16, Pearson’s chi-square test) (see Fig. S4 in the supplemental material). A subset of differences, however, may be attributable to mutations giving rise to the generation or disruption of −10 box motifs, resulting in the emergence or deletion of TSSs. In two recent studies, SNP-driven promoter differences were found to account for over 10% of transcriptional differences between clinical isolates of *M. tuberculosis* (42) and to give rise to strain-specific asRNA transcripts in *M. bovis* (43).

A total of 52 of 219 orthologs which have previously been reported to be differentially expressed between the species (44, 45) are associated with SNP-driven promoter differences (see Table S5 in the supplemental material). For example, *whiB6*, encoding a putative oxidoreductase, has been shown to be highly expressed in *M. bovis* relative to *M. tuberculosis* (44, 45). Closer inspection of our TSS map shows a consensus −10 box motif present upstream of the gene in *M. bovis*, while an insertion event has disrupted this motif in *M. tuberculosis*. Overall, we identified clear correlations between SNP driven-promoter activity and published transcriptional differences between *M. bovis* and *M. tuberculosis*, which will assist in interpreting the molecular basis for their broader phenotypic differences.

## DISCUSSION

Ours is the first study comparing primary transcriptomes across mycobacterial species, and we show that a high level of conservation of 5′ UTRs within CPT orthologs has been maintained between *M. marinum* and *M. bovis*, indicating the potential for a conserved regulatory architecture. Where deviations are observed in 5′ UTR length, we point toward MIRUs as a potential contributor to transcriptional regulation in the *M. tuberculosis* complex. We find evidence of evolutionarily conserved transcriptional attenuation centered on a uORF within the 5′ UTR of *whiB7* and highlight the role that it may play in *whiB7*-mediated antibiotic resistance. The −10 box is the most prominent arbiter of transcriptional initiation, but we also identify a conserved signal at the −35 region of a subset of mycobacterial promoters. Finally, our study results point to SNP-driven promoter differences as a major driver underlying the transcriptional differences between *M. bovis* and *M. tuberculosis*.

Our study was designed to address the influence of differing modes of selection on the primary transcriptome, and recent studies have predicted that there is at least some degree of nonconserved transcriptional noise within certain classes of NCRNA (9). Evolutionary theory posits that many distinctive genome features have their genesis in nonadaptive forces that predominate in the environments of certain lineages (46). Our comparison of *M. marinum* and *M. bovis*, whose genomes have been molded by distinct experiences of natural selection, illustrates that this can be clearly observed within their primary transcriptomes. Previous studies have shown that AT-enriching SNPs are more abundant than expected in the genomes of members of the *M. tuberculosis* complex (35).

It would appear that such an accumulation of SNPs, ineffectively purged by natural selection, has set the stage for an overabundance of promoter sites in the *M. tuberculosis* complex relative to other generalist mycobacteria. Our study further indicated that where such sites exist, the majority are utilized. An underrepresentation of nonfunctional promoter motifs has previously been correlated with translational efficiency and increased growth rate (47), suggesting that an abundance of motifs could have a negative impact on metabolic activity and growth rate. Strong intragenic −10 box motifs could theoretically cause the ribosome to stall at internal sites within genes or could bind RNAP to maintain a local abundance of RNAP molecules close to the pTSS.

The extent to which this noisy transcriptome implies increased regulation by NCRNA is difficult to determine, as our findings argue for the primary transcriptome as a milieu of adaptive and nonadaptive components. However, SNP-driven promoter usage clearly is an important contributor to transcriptional diversity within the *M. tuberculosis* complex and recent studies suggest that double-stranded RNA (dsRNA) plays a major role in gene regulation (48). If this holds true for mycobacteria, then members of the *M. tuberculosis* complex clearly have a greater reservoir from which to initiate transcription than closely related species. Problematically, few reliable methods have emerged for identifying functional NCRNAs and even highly expressed antisense transcripts have been shown to display little evolutionary conservation. Our report shows that this dearth of conservation affects all nonprimary classes of TSS, making prediction of *in silico* function intrinsically difficult. We find that, although a core of intergenic sRNAs is shared, many are associated with 5′ UTRs or may represent misannotated peptides.

The picture emerges of a transcriptome highly conserved in parts, coupled with the potential to generate immense transcriptional diversity. The simplest explanation is that those regions that are hypervariable are passively accumulated, arising from the reduction in the population size of the *M. tuberculosis* complex relative to closely related species of nontuberculous mycobacteria (NTM). An alternative interpretation requires that selective forces are operating to increase the level of transcriptional complexity in the *M. tuberculosis* complex in excess of that of their closest relatives.

Our report predicts that many unique features of a pathogen’s primary transcriptome are merely byproducts of its population history rather than active participants in adaptation to pathogenicity. In any event, consideration of the contributions that nonadaptive neutral forces make in generating transcriptional and regulatory networks seems a necessary prerequisite in order to sort the signal from the noise.

## MATERIALS AND METHODS

Additional details of the experimental procedures are provided in Document S1 in the supplemental material.

### Cell culture and RNA isolation.

*M. bovis* AF2122/97 was grown in roller bottle culture at 37°C in Middlebrook 7H9 medium supplemented with 10% albumin–dextrose catalase (ADC; Difco), 0.05% Tween, and 10 mM pyruvate. *M. marinum* M was grown in roller bottle culture at 30°C in Middlebrook 7H9 medium supplemented with 10% albumin–dextrose catalase (ADC; Difco), 0.05% Tween, and 0.5% glycerol.

Total RNA samples were then isolated using an RNeasy Plus Minikit (Qiagen) in accordance with the manufacturer’s instructions. Samples were quantified using a Nanodrop 1000 spectrophotometer (Thermo Scientific), and the quality of the RNA was assessed by visualization on agarose gels and by measuring the sample’s *A*_260_/*A*_280_ ratio (>1.8 required).

### cDNA library preparation.

TSS sequencing (TSS-seq) cDNA libraries were constructed and sequenced as single-ended, strand-specific reads on a single lane using an Illumina HiSeq 2000 machine by vertis Biotechnologie AG. Standard RNA-seq libraries were also prepared for an Illumina Genome Analyzer IIx platform using RNA from exponential-phase cultures of both species.

### Read mapping and statistical analyses.

Reads were aligned to the reference genomes of *M. bovis* AF2122/97 (NC_002945.3) and *M. marinum* M (NC_010612.1) using Bow tie (49) with the “--best” flag, and biological replicates were processed separately.

The TSS-seq (TEX^+^ and TEX^−^) libraries yielded an average of 12.5 million reads per bioreplicate of *M. bovis* and 13.0 million reads per bioreplicate of *M. marinum*, 80% and 77% of which mapped to the respective genomes. On average, 63% and 51% of the mapped reads, respectively, corresponded to rRNA and were discarded from further analyses. The standard RNA-seq libraries yielded 5.8 million reads on average for *M. bovis* and 16.6 million reads on average for *M. marinum*, 71% and 69% of which mapped to their respective genomes following removal of rRNA reads (~5% and ~24%).

### Interspecies TSS and promoter comparison.

Orthologous genes shared between *M. marinum* and *M. bovis* were retrieved from the Efficient Database framework for Comparative Genome Analyses (EDGAR) (50). Genome-wide TSS maps for both species were generated by assigning all TSSs to at least one of five categories based on their locations relative to annotated genes, in an approach similar to that of Sharma et al. (7). Each TSS was used to define a promoter region 50 nt in length immediately upstream. TSSs associated with orthologous genes were assigned to one of three categories, following a classification scheme used by Kim et al. (11). Promoters were extracted and aligned (51) to the corresponding orthologous genomic regions by the use of ClustalW2. Circular genome representations were created using Circos (52).

### Nucleotide sequence accession number.

The sequencing data described in this report have been submitted to the NCBI gene expression omnibus (GEO) under accession number GSE51881.

## SUPPLEMENTAL MATERIAL

Document S1 Experimental procedures. Download Document S1, DOC file, 0.1 MB

Figure S1 Classification scheme for generating TSS maps. All TSSs were assigned to at least one of five categories, depending on their locations relative to annotated genes. Download Figure S1, JPEG file, 0.1 MB

Figure S2 Motifs identified within the promoters of both species. Download Figure S2, JPEG file, 0.3 MB

Figure S3 Peak heights of TSSs according to promoter motif type in *M. bovis* and *M. marinum*. ****, *P* < 2.2e-16, Pearson’s chi-squared test. Download Figure S3, JPEG file, 0.3 MB

Figure S4 Read depth of *M. tuberculosis* TSS-seq at sites matching TSSs detected only in *M. bovis*. The inset shows the null expectation for the number of randomly selected genomic sites with a minimum peak height of 10 TSS-seq reads. Download Figure S4, JPEG file, 0.2 MB

Table S1 Sequencing statistics and full list of TSS calls in both species, with motifs identified within their promoter regions. (a) Summary of RNA-seq and TSS-seq reads. (b) Full list of orthologous genes identified between *M. marinum* and *M. bovis*. (c) Full list of experimental TSSs identified in *M. marinum*. (d) Full list of experimental TSSs identified in *M. bovis* Table S1, XLS file, 1.7 MB.

Table S2 TSSs shared between *M. marinum* and *M. bovis*. (a) Shared primary TSSs (pTSSs). (b) Shared antisense TSSs (asTSSs). (c) Shared internal TSS (iTSSs). Table S2, XLS file, 0.2 MB.

Table S3 5′ UTR length comparison and misannotated genes in *M. bovis*. (a) Comparison of 5′ UTR lengths of orthologous genes with conserved pTSSs (CPT). (b) Comparison of 5′ UTR lengths of orthologous genes predicted to be misannotated in *M. tuberculosis*. (c) Mycobacterial interspersed repetitive units (MIRUs) located within the 5′ UTRs of genes in *M. bovis*. (d) Shared pTSSs with a conserved TT signal at the −35 region. (e) Orthologous genes with shared transcriptional attenuation patterns in the 5′ UTR. Table S3, XLS file, 0.2 MB.

Table S4 TSSs assigned to intergenic small RNAs previously identified in mycobacteria. Table S4, XLS file, 0.1 MB.

Table S5 Promoter mutations associated with differentially expressed *M. bovis* and *M. tuberculosis* genes. (a) TSSs identified in *M. bovis* which have no matching TSSs in *M. tuberculosis* and promoter sequences mutated between the species. (b) TSSs identified in *M. tuberculosis* which have no matching TSSs in *M. bovis* and promoter sequences mutated between the species. Table S5, XLS file, 0.1 MB.
