# Supplementary material for: Relaxed Selection Drives a Noisy Noncoding Transcriptome in Members of the Mycobacterium tuberculosis Complex
Source: mBio. 2014 Aug 5;5(4):e01169-14. doi: 10.1128/mBio.01169-14 (PMC4128351; doi:10.1128/mBio.01169-14)
Supplement: Document S1 — Experimental procedures. Download [file mbo004141929s1.doc]

**Supplemental Experimental Procedures**

**Cell culture and RNA isolation**

M. bovis AF2122/97 was grown in roller bottle culture at 37°C in Middlebrook 7H9 medium supplemented with 10% albumin-dextrose catalase (ADC, Difco), 0.05% Tween, and 10mM pyruvate. *M. marinum* M was grown in roller bottle culture at 30°C in Middlebrook 7H9 medium supplemented with 10% albumin-dextrose catalase (ADC, Difco), 0.05% Tween, and 0.5% glycerol. 3 ml of cells from mid-log phase cultures (OD = 0.6) were mixed with 6 ml RNAprotect Bacteria Reagent (Qiagen) by vortexing for 5 seconds, incubated for 5 minutes at room temperature, and then centrifuged at 5000 × g for 10 minutes. The supernatant was decanted and any residual supernatant was removed by inverting the tube once onto a paper towel.

Total RNA samples were then isolated using the RNeasy Plus Mini kit (Qiagen) in accordance with the manufacturer's instructions. Samples were quantified using a Nanodrop 1000 spectrophotometer (Thermo Scientific) and the quality of the RNA was assessed by visualization on agarose gels and by measuring the sample's A_260_/A_280_ ratio (>1.8 required).

DNase treatment was carried out using recombinant DNase from USB (USB Molecular Biology Reagents, High Wycombe, UK), and ribosomal RNA (rRNA) was selectively depleted from total isolated RNA using the Ribo-Zero™ bacterial rRNA removal kit (Epicentre Biotechnologies), according to the manufacturer's instructions.

**cDNA library preparation**

TSS sequencing (TSS-seq) cDNA libraries were constructed and sequenced as single-end, strand specific reads on a single lane using the Illumina HiSeq 2000 machine by vertis Biotechnologie AG. For depletion of processed (mono-phosphorylated) transcripts, terminator-5′-phosphate-dependent exonuclease (TEX, Epicentre Biotechnologies) was used. TEX+ RNA was generated by incubation with 1 U of TEX per 1 μg total RNA, and TEX- RNA was generated from total RNA or in buffer alone for 60 mins at 30°C.

TEX+ and TEX- RNA samples were precipitated overnight using a mixture of 2.5 volumes of ethanol and 0.1M sodium acetate (pH 6.5), and treated with 1 U of tobacco acid pyrophosphatase (TAP, Epicentre Biotechnologies) at 37°C for 1 hour.

RNA samples were fragmented using divalent cations (Fragmentation Buffer, Ambion) at 70°C for 5 min, and then precipitated with ethanol. RNA was then polyadenylated using poly(A) polymerase, following which an RNA adapter was ligated to the 5'-(P). First-strand cDNA synthesis was performed with an oligo(dT) adapter primer and M-MLV reverse transcriptase. The resulting cDNA was amplified by PCR with a high fidelity DNA polymerase to a concentration of between 20 and 30 ng/μl.

Standard RNA-seq libraries were also prepared for the Illumina Genome Analyzer IIx using RNA from exponential phase cultures of both species. For these libraries, first strand cDNA synthesis was performed in a reaction using the Super Script II reverse transcriptase (200 U), random hexamer primers (3 µg) and dNTPs (500 µM). Reaction components were then removed using Illustra MicroSpin G-50 columns (GE Healthcare Biosciences, Pittsburg, PA, USA).

dUTP containing second strand cDNA was generated with DNA Polymerase I (50 U) and RNase H (2 U) in a 1 times 2nd strand buffer (Invitrogen, Carlsbad, CA, USA) also containing 300 µM of a mixture of dATP, dCTP, dGTP, and dUTP, but not dTTP. Products were then further processed and end repaired, with the addition of a single A to the 3′ end and ligation of indexed adapters. Samples were multiplexed by the addition of 6 nt barcoded Illumina-compatible adapters.

Libraries were sized selected on 2.5% TAE agarose gels. Library material was isolated from gel slices using the QiaQuick MinElute Gel Extraction kit (Qiagen). Prior to library amplification, the dUTP-containing second strand was removed via digestion with Uracil DNA Glycoylase (1 U) (Bioline). Purified libraries were quantified using a Qubit™ fluorometer (Invitrogen) and a Quant-iT™ double-stranded DNA High-Sensitivity Assay Kit (Invitrogen). Clustering and sequencing of the material was carried out as per the manufacturer's instructions – v2 Single Read Cluster Kits and v3 SBS kits (Illumina) were utilized for all sequencing.

**Read mapping and statistical analyses**

Reads were aligned to the reference genomes of M. bovis AF2122/97 (NC_002945.3) and *M. marinum* M (NC_010612.1) using Bowtie (1) with the “--best” flag, and biological replicates were processed separately.

The TSS-seq (TEX+ and TEX-) libraries yielded an average of 12.5 million reads per bio-replicate of *M. bovis* and 13.0 million reads per bio-replicate of *M. marinum*, of which 80% and 77% mapped to the respective genomes. On average, 63% and 51% of mapped reads, respectively, corresponded to rRNA and were discarded from further analyses. The standard RNA-seq libraries yielded 5.8 million reads on average for *M. bovis* and 16.6 million reads on average for *M. marinum*, of which 71% and 69% mapped to their respective genomes following removal of rRNA reads (~5% and ~24%).

Genome alignment files were stored in BAM and in GFF (generalized feature format) format files and visualized using the Artemis genome browser (2). Orthologous genes shared between *M. marinum* and *M. bovis* were retrieved from the *Mycobacterium* project of EDGAR online tools (3). Data analyses and statistical tests were carried out using scripts written for the Python v2.7.5 and R v3.0.2 programming languages. RPKM (reads per kilobase per million mapped reads) values were calculated according to *Mortazavi* et al. (2008) (4).

For TSS calling, the genome coordinates of the 5' ends of all uniquely mapped TEX+ library reads were determined, and a custom Perl script was implemented to determine the TEX+ and TEX- library 5' read depth at each genomic position. A candidate TSS was called when a genomic position was encountered with a minimum TEX+ reads to TEX- reads ratio of 2:1, with at least 20 supporting TEX+ reads. If more than one such site existed within 50 nt, then the site with greatest TEX+ read depth was considered the candidate TSS. Only candidate TSSs supported by both biological replicates (with ± 10 nt tolerance) were retained.

These analyses resulted in a final number of 6500 TSS and 6331 TSS calls for the *M. bovis* and *M. marinum* genomes, respectively. TSS peak height was calculated as the maximum TEX+ library read depth within 50 nt downstream of the annotated TSS position.

Functional categories of genes in *M. tuberculosis* were retrieved from Tuberculist (5) and mapped to their respective orthologous genes. Alignments of 5' UTRs were carried out using ClustalW2 (6). To determine 5' UTRs showing transcriptional attenuation patterns, the effective RPKM of the 5' UTR {1} was calculated, as defined by the primary TSS and the start codon of the associated gene, and then compared with the measured RPKM of the coding sequence (CDS) {2}. Genes showing a high ratio of {1}:{2} were considered to have RNA-seq patterns consistent with transcriptional attenuation in the 5' UTR.

**TSS & promoter annotation**

Genome-wide TSS maps for both species were generated by assigning all TSSs to at least one of 5 categories based on their locations relative to annotated genes, in a similar approach to that of Sharma *et al*. (7) (**Figure S1**). Primary and alternative TSSs were those located within 500 nt of the start codon of a gene on the same strand, with the former being those the largest TEX + peak height. Internal TSSs were those located between the start and end codons of a gene on the same strand, while antisense TSS were defined as those on the opposite strand to to the coding regions or the 5' UTRs of genes. Intergenic TSSs were those which could not be assigned to any other category.

A comprehensive recent bioinformatic survey by DeJesus *et al.* (2013) (8) identified a set of 269 genes in *M. tuberculosis* which are likely to have misannotated start codon positions. The start codon positions of the *M. bovis* orthologs were adjusted accordingly during the TSS classification process.

Each TSS was used to define a promoter region 50 nt in length immediately upstream. Motifs from both species were visualised using MEME (9). Statistical analyses of CPT promoter sequences were carried out and visualised with pLogo (10), using a background dataset of randomly selected 50 nt regions from both genomes.

**Inter-species promoter/TSS comparison**

TSSs associated with orthologous genes were assigned to one of three categories, following a classification scheme used by Kim *et al.* (2012) (11); conserved promoter with matching TSS (CPT), conserved promoter with no matching TSS (CPNT) or orphan promoter (OP). Promoters were extracted and aligned using ClustalW2 (4) to the corresponding region of genome, containing the orthologous gene and 500 nt either side, from the other species.

If a matching TSS was detected at the 3' end of the alignment (± 10 nt), then the promoter of the matching TSS was aligned back to the corresponding genomic region from the first species. If the 3' of this alignment matched the first TSS, then both promoters were classified as CPT. If no corresponding TSS was detected in the first alignment, then the matching genomic region was aligned back to the first species, and if it matched with the original promoter, then that promoter was classified as CPNT. If it did not match the original promoter, then that promoter was classified as OP. Promoter mutations between *M. bovis* and *M. tuberculosis* were identified using the same process.

For comparison with *M. tuberculosis* TSS sequencing, data was retrieved from EBI Array Express (E-MTAB-1616). To determine the maximum peak height, the 5' read depth at each genomic coordinate was measured for all 3 biological replicates and the maximum value was taken. To calculate the null expectation, 3692 genomic sites (equal to the number of TSS detected only in *M. bovis*) were randomly selected a total of 1,000,000 times, and the proportion of sites associated with a maximum peak height of at least 10 reads was calculated. The actual value for sites matching *M. bovis* TSSs was then compared with the expected null distribution.

To map intergenic sRNAs, the sequence of the sRNA was extracted from the approporiate reference genome and submitted to a BLASTn search of the *M. bovis* and *M. marinum* genomes. If a match with e value < 1e-5 was found, the matching region was interrogated for the presence of an experimentally-defined TSS in both species.

**Supplemental references**

1. **Langmead B, Trapnell C, Pop M, Salzberg SL**. 2009. Ultrafast and memory-efficient alignment of short DNA sequences to the human genome. Genome Biol **10**:R25.

2. **Carver T, Harris SR, Berriman M, Parkhill J, McQuillan JA**. 2012. Artemis: an integrated platform for visualization and analysis of high-throughput sequence-based experimental data. Bioinformatics **28**:464–469.

3. **Blom J, Albaum SP, Doppmeier D, Pühler A, Vorhölter F-J, Zakrzewski M, Goesmann A.** 2009. EDGAR: a software framework for the comparative analysis of prokaryotic genomes. BMC Bioinformatics **10:**154.

4. **Mortazavi A, Williams BA, McCue K, Schaeffer L, Wold B.** 2008. Mapping and quantifying mammalian transcriptomes by RNA-Seq. Nat Meth **5:**621-628.

5. **Lew JM, Kapopoulou A, Jones LM, Cole ST**. 2011. TubercuList–10 years after. Tuberculosis **91**:1–7.

6. **Larkin MA, Blackshields G, Brown NP, Chenna R, McGettigan PA, McWilliam H, Valentin F, Wallace IM, Wilm A, Lopez R**. 2007. Clustal W and Clustal X version 2.0. Bioinformatics **23**:2947–2948.

7. **Sharma CM, Hoffmann S, Darfeuille F, Reignier J, Findeiß S, Sittka A, Chabas S, Reiche K, Hackermüller J, Reinhardt R**. 2010. The primary transcriptome of the major human pathogen Helicobacter pylori. Nature **464**:250–255.

8. **DeJesus MA, Sacchettini JC, Ioerger TR**. 2013. Reannotation of translational start sites in the genome of Mycobacterium tuberculosis. Featur. Rep. Tuberc. Community Annot. Proj. Jamboree Held Va. Tech USA March 7-8 2012 **93**:18–25.

9. **Bailey TL, Boden M, Buske FA, Frith M, Grant CE, Clementi L, Ren J, Li WW, Noble WS**. 2009. MEME SUITE: tools for motif discovery and searching. Nucleic Acids Res. **37**:W202–W208.

10. **O’Shea JP, Chou MF, Quader SA, Ryan JK, Church GM, Schwartz D**. 2013. pLogo: a probabilistic approach to visualizing sequence motifs. Nat. Methods **10**:1211–1212.

11. **Kim D, Hong JS-J, Qiu Y, Nagarajan H, Seo J-H, Cho B-K, Tsai S-F, Palsson BØ**. 2012. Comparative analysis of regulatory elements between Escherichia coli and Klebsiella pneumoniae by genome-wide transcription start site profiling. PLoS Genet. **8**:e1002867.
