# Supplementary figures and images for: Relaxed Selection Drives a Noisy Noncoding Transcriptome in Members of the Mycobacterium tuberculosis Complex
Source: mBio. 2014 Aug 5;5(4):e01169-14. doi: 10.1128/mBio.01169-14 (PMC4128351; doi:10.1128/mBio.01169-14)

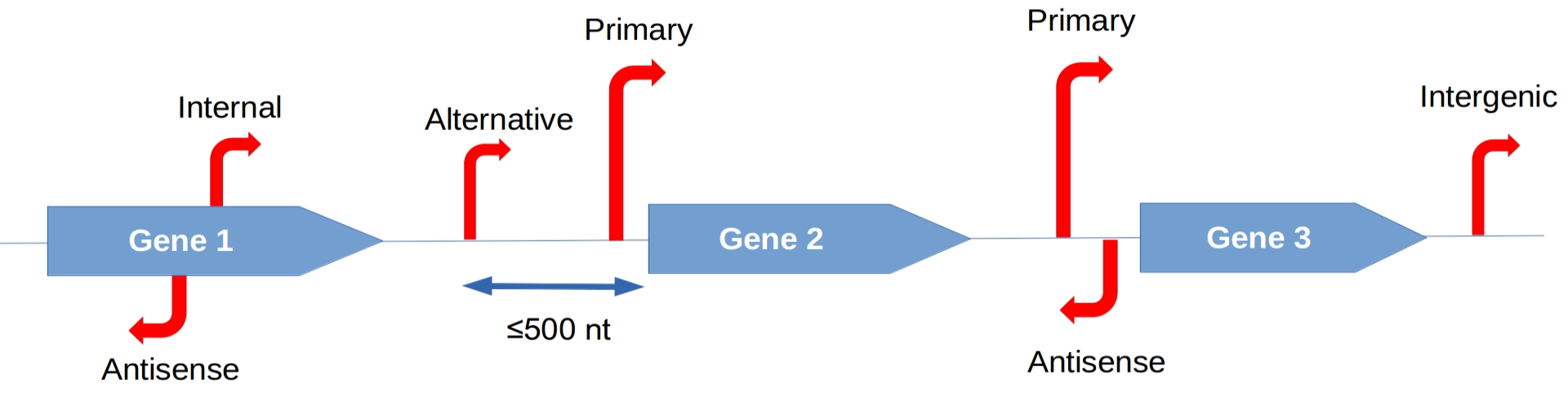

Supplement: Figure S1 — Classification scheme for generating TSS maps. All TSSs were assigned to at least one of five categories, depending on their locations relative to annotated genes. Download [file mbo004141929sf01.jpg]

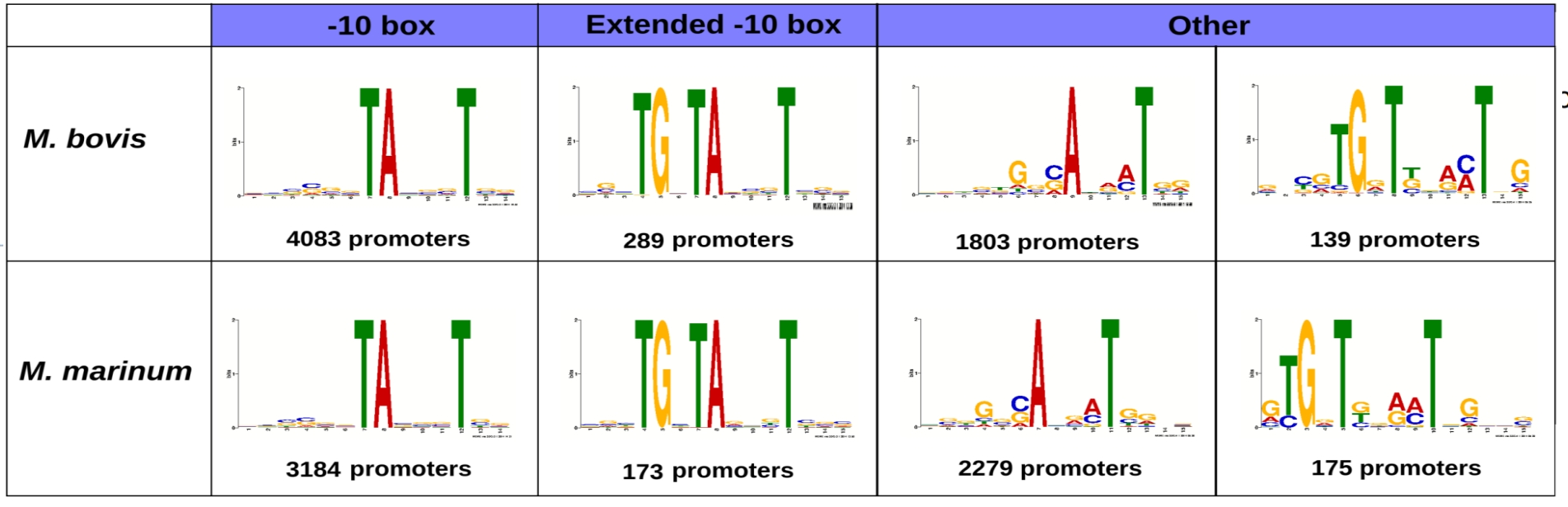

Supplement: Figure S2 — Motifs identified within the promoters of both species. Download [file mbo004141929sf02.jpg]

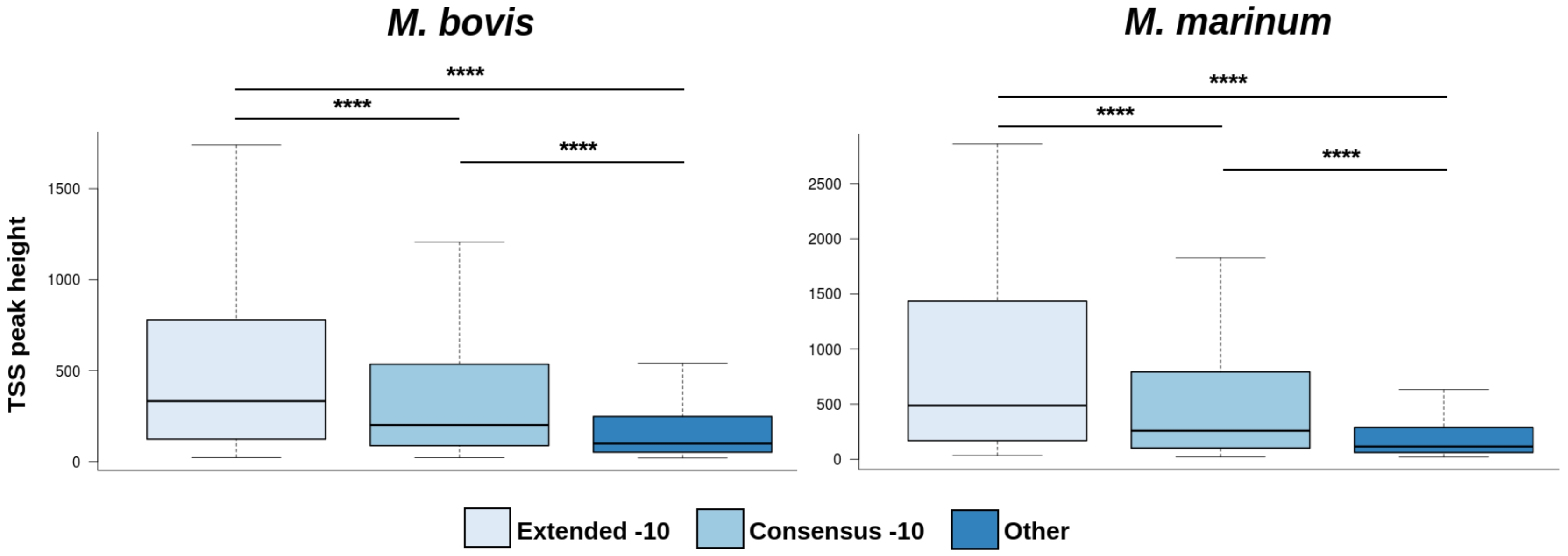

Supplement: Figure S3 — Peak heights of TSSs according to promoter motif type in M. bovis and M. marinum. ****, P < 2.2e-16, Pearson’s chi-squared test. Download [file mbo004141929sf03.jpg]

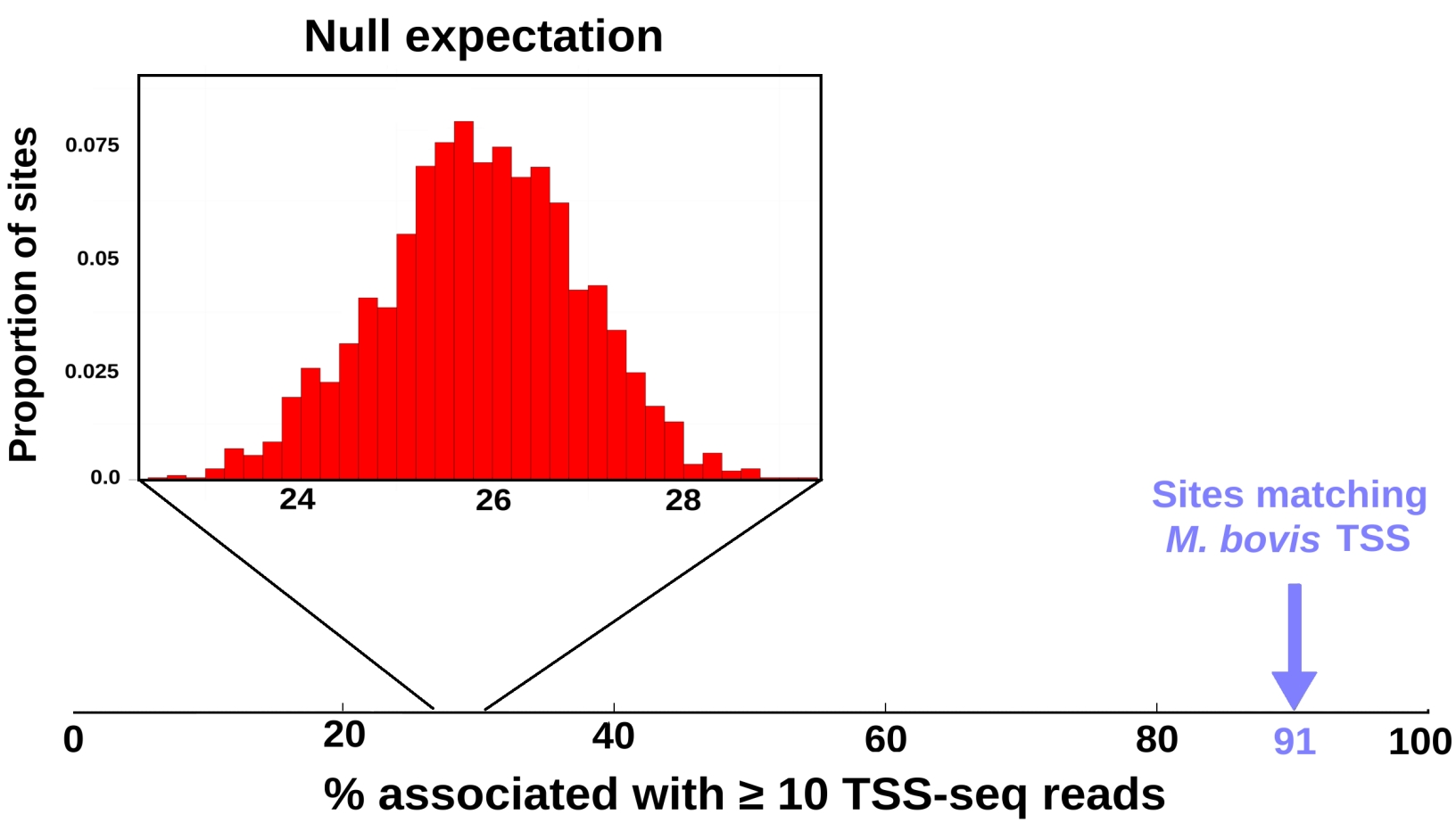

Supplement: Figure S4 — Read depth of M. tuberculosis TSS-seq at sites matching TSSs detected only in M. bovis. The inset shows the null expectation for the number of randomly selected genomic sites with a minimum peak height of 10 TSS-seq reads. Download [file mbo004141929sf04.jpg]
